# Effects of Germination on the Fatty Acid, Folate, and Mineral Levels in Quinoa

**DOI:** 10.1002/fsn3.71269

**Published:** 2025-11-26

**Authors:** Boyuan Li, Ting Wang, Muhui Wang, Yueying Zhang, Pak‐Ho Wong, Yi Wang

**Affiliations:** ^1^ Department of Chemistry, Faculty of Sciences Hong Kong Baptist University Hong Kong P.R. China; ^2^ College of Food Science and Engineering Ocean University of China Qingdao P.R. China

**Keywords:** brain health, GC–MS, germination, HPLC, nutrients, quinoa

## Abstract

This study investigated the nutritional changes in germinated quinoa and its relevance to early brain health. The contents of fatty acid (FA), folate, protein, and minerals in quinoa under different germination times were determined. Firstly, GC–MS results showed that germination significantly changed the composition and content of FAs. Additionally, the HPLC results showed that the folate content increased to 349.63 mg/100 g after 72 h, which is sufficient to meet the needs of early brain development in babies. More importantly, the iron, zinc, and copper contents increased by 75.90%, 6.38%, and 13.58% respectively after 48 h of germination. The higher mineral contents can adequately fulfill the daily requirements for early brain health. In summary, this study proves that germination is an effective way to regulate the nutrient levels of quinoa. Furthermore, this study is valuable for improving other grains and shows significant promise for the future development of functional foods.

## Graphical abstract

This study analyzed the change of the key nutrients in quinoa during its short‐term germination process. The results showed that germination significantly increases the content of folate, protein, and trace minerals, thereby maximizing its nutritional properties for promoting neural development. This study demonstrated that 48 h of germination is a prospective method to transform quinoa into a special food for protecting brain health without genetic modification or fortification.

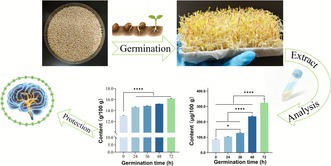

## 1 Introduction

The energy and nutrients necessary for early fetal brain development heavily rely on the mother's dietary intake and its conversion (Mou et al. 2023). Malnutrition during the mother's pregnancy or infancy can severely hinder fetal brain development, affecting cognition, learning, and behavior. Currently, around 200 million children under the age of five face significant challenges related to unbalanced diets and malnutrition worldwide, which extremely limits their developmental potential (World Health Organization et al. 2023). Therefore, how to improve the nutritional status of these children has become a critical global concern. Under this situation, exploring dietary supplements that promote brain health can effectively leverage food resources to support the brain development of fetuses and infants, ultimately preventing brain damage. Lipids, particularly unsaturated fatty acids (UFAs), are vital for the formation of brain structure and functions, with about 50% of the brain's FAs being polyunsaturated fatty acids (PUFAs), crucial for brain nerve development (Ryan et al. 2013; van de Rest et al. 2012). Additionally, various micronutrients, especially vitamins and trace minerals, are also vital in the early stages of brain development, serving as essential components for neurodevelopment (Cunnane and Crawford 2014; Mattei and Pietrobelli 2019).

Germination is the biological process in which seeds activate their internal enzyme systems under suitable conditions, enabling the radicle to emerge from the seed coat and begin growing (Dikbaş et al. 2023). Germination not only improves the digestibility of grains but also optimizes their nutritional ratio through biotransformation, improving human metabolism regulation and immune function (Lan et al. 2023). The metabolic activity within the seeds increases significantly during this process, leading to the breakdown of starch into easily digestible sugars, promoting the hydrolysis of fats into free FAs, and catalyzing the conversion of proteins into free amino acids (Liu et al. 2022). Fat serves as the primary energy source during seed germination. It is mainly present as triglycerides, which are enzymatically converted into glycerol and FAs (Liu et al. 2023). Therefore, during the early stages of germination, the essential energy required for seed growth is provided by the oxidative metabolism of FAs. The related study indicated that following quinoa germination, the levels of linoleic acid and linolenic acid increased (Jan et al. 2018). In addition, another research also confirmed that germination is an effective way to change the contents of various FAs in quinoa (Pandya et al. 2021). This change could influence the formation of membrane structures during brain development by modulating lipid metabolism (Deoni et al. 2018), which is especially significant in research focused on early brain development of infants.

Quinoa, a pseudo‐cereal originating from South America, is abundant in high‐quality protein, dietary fiber, and a range of vitamins and minerals (Chen et al. 2023). Quinoa has a low glycemic index and is gluten‐free, making it a better option for enhancing satiety and promoting digestive health compared to grains like wheat and corn. It is often celebrated as a “complete nutritional superfood” due to its exceptional nutritional richness and balance (Chen et al. 2023). The Food and Agriculture Organization (FAO) of the United Nations has identified quinoa as one of the most promising plants that can meet basic human nutritional needs (Nowak et al. 2016). In addition, quinoa also contains ingredients with pharmacological effects, such as saponins and polyphenols (Lan et al. 2023) giving it high application value in agriculture, medicine, and other fields.

Vitamins are a class of trace organic compounds necessary for maintaining normal physiological functions of the human body. The folate, in particular, is crucial for DNA synthesis, neural tube closure, and myelination, which are essential for early brain development (Sable et al. 2011). However, folate cannot be synthesized by the human body alone to meet daily needs and must be obtained through additional intake. Furthermore, folate deficiency can lead to abnormal neuronal differentiation, an increased risk of neural tube defects, delayed myelination, and cognitive impairments, leading to irreversible damage to neurodevelopment from the fetal stage through infancy (Kang et al. 2019; Kim et al. 2018). Therefore, adequate folate intake is extremely important for brain health from the fetal stage through infancy.

Trace minerals are a type of inorganic elements that the human body requires in very small amounts but are necessary to maintain normal physiological functions. They are also vital for infants' early brain health. Their optimal concentration and bioavailability work together to regulate essential processes like neuronal differentiation, synapse formation, and the intricate development of neural networks (Cioffredi et al. 2024). For brain health, trace elements such as iron (Fe), iodine (I), zinc (Zn), copper (Cu), and selenium (Se) et al. are important; deficiencies in these can lead to widespread and often irreversible neurodevelopmental disorders (Shayganfard 2022). Among them, Fe, Zn, Cu, and Se are particularly important, and their deficiency can lead to extensive and irreversible neurodevelopmental disorders (Prado and Dewey 2014). For example, Fe as a component of hemoglobin, ensures oxygen transport and is also an essential cofactor for the metabolism of multiple neurotransmitters (McCann et al. 2020). Its deficiency can seriously impair cognitive, motor, and emotional development in babies. Additionally, Zn is necessary for DNA synthesis and repair, neurogenesis, hippocampus‐dependent learning, and memory (Willekens and Runnels 2022). A deficiency in Zn can impair neural precursor cell proliferation, neuronal morphogenesis, and synaptic plasticity, affecting cognition and behavior. Furthermore, Cu is a core component of multiple key enzymes, such as cytochrome C oxidase, superoxide dismutase, and dopamine β‐hydroxylase (Tarnacka et al. 2021). Its insufficiency can result in abnormal neuronal development and myelination defects, while excess Cu is neurotoxic. Finally, Se exerts a powerful antioxidant effect through selenoproteins, protects the vulnerable developing brain from oxidative damage, and plays a role in the regulation of thyroid hormone metabolism (Huang et al. 2025). The lack or surplus of these trace elements tends to interact and often occurs together. Their effects are interconnected and jointly influence the determination of the fate of neural stem cells, axon guidance, synaptic efficacy, and neuroprotective mechanisms (Moreno‐Fernandez et al. 2020). Due to the strict time and space windows and irreversibility of early brain development, ensuring an adequate and balanced supply of these key trace elements is of decisive significance.

Consequently, as a natural source of bioactive substances, the germinated products of quinoa not only have comprehensive nutrition but also exhibit dynamic changes in their functional components, offering a unique perspective for studying their connection with early brain development. While some studies have begun to explore quinoa sprout products, their analyses are relatively basic. For instance, the changes in FAs (Vera et al. 2019), polyphenol (Bhinder et al. 2021), and vitamin variations (Darwish et al. 2021) during germination in quinoa.

Currently, a comprehensive study on quinoa germination is still lacking. Therefore, this study analyzed the changes in key nutrients during quinoa sprouting and reported for the first time the potential of sprouted quinoa as a special food to enhance early brain health. To obtain dynamic changes in quinoa's nutritional components, we controlled the germination time to obtain quinoa products with varying germination durations at first. Then the FAs in quinoa were extracted using Soxhlet extraction, after which the crude extract was methylated for GC–MS analysis of its FA composition. The folate content in the products was measured by HPLC‐UV. The protein content of the sprouted products was determined by the Kjeldahl method. Finally, ICP‐OES was used to analyze the mineral element levels in the products.

## 2 Materials and Methods

### 2.1 Materials

Quinoa was purchased from a local supermarket in Hong Kong. Sodium dihydrogen phosphate anhydrous, disodium hydrogen phosphate anhydrous, sodium ascorbate, β‐mercaptoethanol, sodium methoxide, formic acid, methanol, folate (≥ 98%), and neutral protease (BR) were purchased from Macklin Biochemical Technology Co. Ltd. (Shanghai, China). Sodium hypochlorite solution and thermostable α‐amylase (BR) were obtained from Aladdin Biochemical Technology Co. Ltd. (Shanghai, China). Normal rat serum was purchased from Abbkine Scientific Co. Ltd. (Wuhan, China). *N*‐hexane (HPLC grade) was bought from Anaqua Global International Inc. Limited (Hong Kong). Acetonitrile (ACN, HPLC grade) was purchased from International Laboratory USA (San Francisco, USA). F.A.M.E. Mix was bought from Sigma‐Aldrich Trading Co. Ltd. (Shanghai, China). The other reagents used, such as hydrochloric acid (HCl) and sodium hydroxide (NaOH), were purchased from Sinopharm Chemical Reagent Co. Ltd. (Shanghai, China). Unless otherwise specified, all chemicals used were of analytical grade.

### 2.2 Germination of Quinoa

The quinoa sprouting process was conducted as per the modified method in the previous literature (Donkor et al. 2012). After washing and drying the quinoa, they were soaked in sodium hypochlorite solution (w/v, 2.5%) for 5 min for disinfection. Afterwards, the seeds were soaked in deionized (DI) water to initiate germination at 25°C for 4 h. The soaked quinoa seeds were then taken out, excess water was drained, and they were placed in a temperature‐controlled incubator at 22°C in the dark for germination. DI water was sprayed regularly, maintaining a relative humidity of 95%–100%. Germination periods of 24, 36, 48, and 72 h were selected to evaluate the impact of different germination times on the nutritional components of quinoa. The quinoa that had not germinated (0 h) served as the control group. After germination, all samples were rinsed three times with DI water and freeze‐dried for subsequent experiments.

### 2.3 Determination of FA Content by GC–MS

#### 2.3.1 Soxhlet Extraction of Crude Fats

Soxhlet extraction is a method for the extraction of soluble substances, especially fats, from solid samples by using the principle of continuous solvent reflux and siphon circulation (Barde et al. 2018). This method can achieve continuous, efficient, and gentle extraction of samples by organic solvent. The freeze‐dried quinoa was ground into powder and dried in an oven to constant weight. Then, 5 g of the powder was weighed, packaged in oily filter paper, and placed in a Soxhlet extractor. An appropriate amount of *n*‐hexane was added to the extraction device and extracted at 80°C for 12–16 h. After the extraction, the sample was taken out and heated to reflux twice again to extract the fat remaining on the tube. Finally, a vacuum rotary evaporator was used to evaporate the excess solvent at 40°C to obtain the crude fat. The obtained oil sample was weighed, and the crude fat content of the sprouted quinoa was calculated.

#### 2.3.2 Methylation of FAs

Free FAs cannot be directly analyzed by GC–MS and must be converted into fatty acid methyl esters (FAMEs) through a methylation reaction. This is because free FAs have high polarity and high boiling points, making it difficult to fully vaporize and effectively separate them directly in a GC column (Yu et al. 2016). While the FAMEs obtained after methylation have significantly reduced polarity and boiling points, making it easier to achieve good separation on a GC column and produce more stable fragment ion spectra, this greatly improves the sensitivity of detection as well as the accuracy of qualitative and quantitative analysis. Referring to the method of previous research, the crude fat extract was modified using the sodium methoxide methylation method (Petrović et al. 2010). Specifically, 50.0 μL of quinoa fat extract was added to a centrifuge tube, and 2.0 mL of sodium methoxide‐methanol solution (0.5 M) was added to react at 55°C for 1.5 h, and the tube was shaken and mixed for 5 s every 15 min. After the reaction, 2.0 mL of saturated sodium bicarbonate solution and 3.0 mL of *n*‐hexane were added, mixed, and centrifuged at 4000 rpm for 15 min. After centrifugation, the supernatant was filtered with a filter membrane (0.22 μm) and injected into a sample bottle waiting for GC–MS testing. The untested samples were stored at 4°C.

#### 2.3.3 GC–MS Measurements

GC–MS is the most commonly used method for FA detection due to its extremely high accuracy and sensitivity. In this study, the samples were tested using Gas Chromatograph with Mass Selective Detector (Agilent 6890N, CA, USA). The parameters of GC–MS were adjusted according to the previous method (Nemzer and Al‐Taher 2023). Specifically, the injection port temperature was set to 270°C. The temperature program was as follows: the initial temperature was 80°C for 10 min, then increased to 180°C at 10°C/min and maintained for 15 min. Next, the temperature was increased to 200°C at 2°C/min and held for 10 min, and finally, it was increased to 240°C at a rate of 3°C/min and maintained for 10 min. The flow rate was 1.0 mL/min and the injection volume was 1.0 μL. The standard curve was drawn using the FAME mixed standard. Specifically, 100.0 mg of the mixed standard was dissolved in *n*‐hexane to prepare a standard solution (2.0 mg/mL), and the standard solutions of 1000.0, 500.0, 250.0, 125.0, 62.5, and 31.3 μg/mL were prepared by the twofold dilution method. The standard curve was drawn with the concentration of the standard solution as the horizontal axis and the chromatographic peak area as the vertical axis. Under the same conditions, the methyl esterification products of fat extract were measured.

### 2.4 Determination of Folate by HPLC‐UV

#### 2.4.1 Extraction of Folate From Quinoa

The extraction of folate was carried out using a method from the previous literature with some modifications (Yazynina et al. 2008). First, a folate extract solution (50.0 mM) was prepared by accurately weighing 2.5 g of anhydrous sodium dihydrogen phosphate and 0.6 g of anhydrous sodium hydrogen phosphate, along with 5.0 g of ascorbic acid sodium salt (1.0%, m/v) and 0.5 mL of β‐mercaptoethanol (0.1%, v/v). Next, 400.0 mL of DI water was added to dissolve the mixture, and the pH was adjusted to 6.5 using 1 M HCl and 1 M NaOH, before bringing the total volume to 500.0 mL. For extraction, 50.0 mg of dried sample powder was placed into a 2.0 mL centrifuge tube and added 1.0 mL of the folate extract solution. The mixture was heated at 100°C for 10 min and then quickly cooled to room temperature in an ice bath. Next, 20.0 μL of α‐amylase was added to the mixture, which was thoroughly mixed and incubated at 37°C for 40 min. After that, it was boiled for 10 min and cooled again. Then, 150.0 μL of protease (40.0 mg/mL) was added as well as mixed, and the mixture was incubated in a 37°C incubator for 1 h. After incubation, the mixture was boiled for 10 min, then quickly cooled to room temperature, and centrifuged for 20 min (14,000 rpm, 4°C). The supernatant was transferred to a new centrifuge tube, and 50.0 μL of rat serum was added. This mixture was incubated at 37°C for 4 h, then boiled for 10 min to inactivate the enzyme. The solution was cooled to room temperature before being centrifuged again for 20 min (14,000 rpm, 4°C). Finally, the supernatant was aspirated and placed in a brown injection bottle for folic acid content analysis.

#### 2.4.2 Determination of Folate Content by HPLC‐UV

The folate contains specific conjugated double bond systems in its molecular structure. When the electrons in these conjugated systems absorb ultraviolet or visible light at specific wavelengths, energy level transitions occur, resulting in characteristic absorption spectra. The folate usually has a maximum absorption peak around 280–290 nm due to its benzene ring structure (Araujo et al. 2012). Therefore, in this study, the UV wavelength of 280 nm was selected to obtain the maximum UV absorption of folate. The samples were tested using HPLC with a Variable UV Detector (Agilent 1100, CA, USA). The HPLC parameters were based on a previous study with some modifications (Riaz et al. 2019), and an Alltech C18 Column (Grace, USA) was applied. The mobile phases A and B were formic acid in water (0.1%, v/v) and formic acid in ACN (0.1%, v/v), respectively. The initial mobile phase B was 5% and the flow rate was 1.0 mL/min. The proportion of mobile phase B increased linearly from 5% to 9% over 2 min. In the next 5.9 min, the proportion of phase B increased to 9.5%, and then it sharply rose to 20% in 0.3 min. After maintaining 20% for 3 min, the proportion of phase B decreased to 5% in 0.2 min and was held for 3 min, with an equilibrium time of 2 min.

### 2.5 Protein Content Determination

The crude protein content in quinoa sprout products was determined using a fully automatic Kjeldahl nitrogen analyzer (Hanon K1160, China). Specifically, 3.0 g of sample powder, dried to constant weight, was tested for N content, and the crude protein content in the sample was calculated using the conventional conversion factor of 6.25.

### 2.6 Minerals Detection

The determination of minerals in the quinoa followed the method in GB 5009.268‐2016 and was performed quantitatively using an inductively coupled plasma optical emission spectrometer (Agilent Technologies 5110 ICP‐OES/ICP‐MS NexlON, USA).

### 2.7 Statistical Analysis

Unless otherwise specified, all the experiments were independently repeated at least three times in parallel and were expressed in the form of the mean ± standard deviation. Statistical analysis of experimental data was performed by SPSS 25.0 software (*p < 0.05, **p < 0.01, ***p < 0.001, ****p < 0.0001).

## 3 Results

### 3.1 Changes in FAs

#### 3.1.1 Changes in Crude Fat Content of Sprouted Quinoa

**FIGURE 1:**
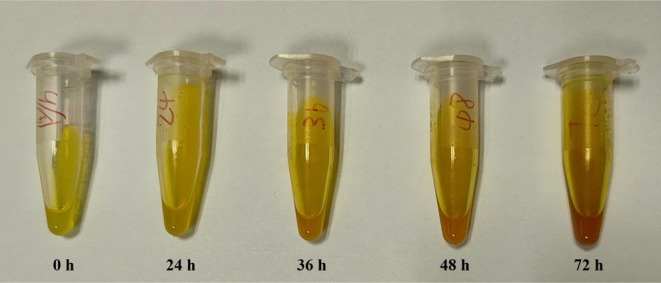
Crude fat extract of quinoa at different germination times.

The crude fat extracted from sprouted quinoa samples using Soxhlet extraction is shown in Figure 1. It can be clearly observed that the color of the extract gradually deepens with the extension of germination time. This may be caused by the oxidation of natural pigments and other substances due to the high temperature of the reaction process. The extraction rate of crude fat is 3.22%–4.33%, which is similar to the results of a related study (Jan et al. 2018) and proves that the extraction of FAs is successful.

**FIGURE 2:**
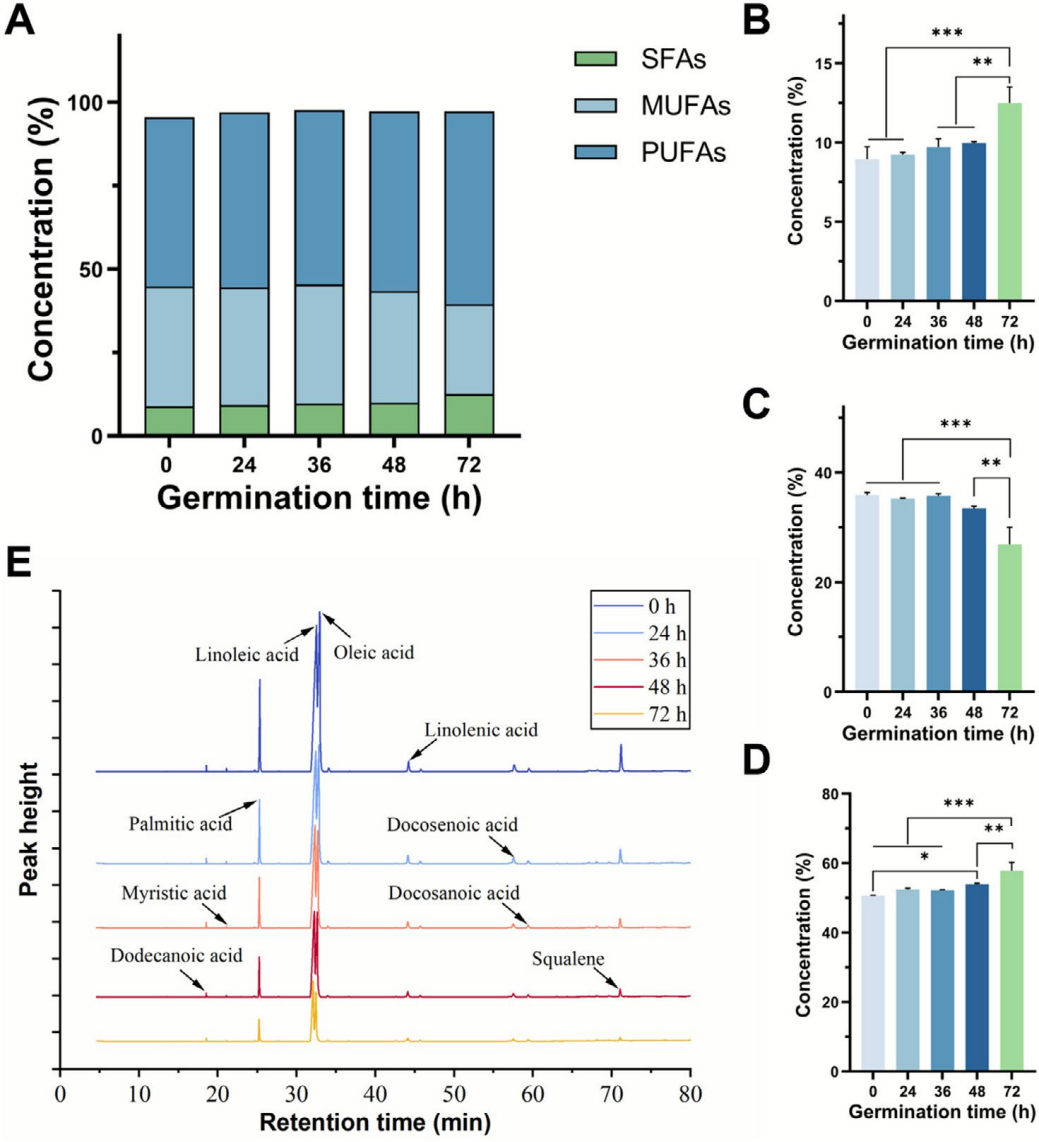
(A) Overall concentration changes in FAs, the concentration changes of (B) SFAs, (C) MUFAs, and (D) PUFAs; (E) GC–MS spectra of crude fat extracts from different germination times. (*p < 0.05, **p < 0.01, ***p < 0.001, ****p < 0.0001).

As shown in Figure 2A–D, the composition of crude fat at different germination times was analyzed. During germination, the proportions of polyunsaturated fatty acids (PUFAs) and saturated fatty acids (SFAs) increased from 50.66% and 8.95% to 57.89% and 12.50%, respectively. The content of monounsaturated fatty acids (MUFAs) decreased from 35.92% to 26.94%. Furthermore, it can be observed that there is no significant difference in the proportion of various FAs during the first 24 h of germination. This is because quinoa absorbs water to break its dormant state at this stage, activating various enzyme systems and starting to decompose the stored macromolecules (Xu et al. 2017). The respiration is not strong at this stage, so the demand for fat is low. With the supply of water, energy and nutrients, the cells at the tip of the radicle begin to divide and elongate rapidly and break through the seed coat after 24 h. Subsequently, the radicle continues to elongate, and the initial root hairs begin to differentiate in the area near the tip of the radicle (Jiang et al. 2025). Therefore, between 24 and 72 h, the seed's energy demand increases rapidly, leading to significant changes in fat contents.

#### 3.1.2 GC–MS Results

The GC–MS spectra are shown in Figure 2E, where the areas of the characteristic peaks for different FAMEs significantly decreased as the germination time increased. This result further proves that changes in FA content of quinoa during germination are due to the consumption of fat as an energy source. The specific data are listed in Table 1. To further explore the changes in FAs, we analyzed six major FAs with contents larger than 1.00%. Obviously, during the germination process, the consumption of fat is proportional to the germination time. From top to bottom, the amount of the six FAs decreased by 80.21%, 68.96%, 59.94%, 67.85%, 64.41%, and 45.19%, respectively. This decrease in FA contents was similar to the results of previous studies (Lan et al. 2023). It is worth noting that at 24 h, the content of linolenic acid was almost not significantly reduced, while the content of linoleic acid decreased quickly by 19.99%. As representatives of omega‐3 (ω‐3) and omega‐6 (ω‐6) FAs, the ratio of linoleic acid to linolenic acid decreased from 12.8:1 to 10.5:1. Studies have shown that the ω‐6 and ω‐3 fats consumed by the human body should have a certain ratio (Schipper et al. 2025). This is because excessive intake of ω‐6 FAs, especially linoleic acid, its oxidative metabolites can promote inflammatory responses in the brain, interfere with neural signal transmission, and increase the risk of neurodegenerative diseases (Yan et al. 2025). In addition, another study found that a high proportion of ω‐6 FAs in breast milk was significantly associated with decreased movement level, cognitive scores, and verbal IQ in infants and young children (Sullivan et al. 2023). Therefore, an adequate and balanced intake of ω‐3 FAs is essential, as it can significantly improve baby's cognitive function and help to reduce the risk of neurodevelopmental disorders. The anti‐inflammatory effects of ω‐3 FAs can protect neurons from oxidative damage and provide an optimized environment for neurotransmitter regulation and synaptic growth. Therefore, we believe that short‐term germination may be a distinctive strategy to prevent abnormal brain development and enhance neurological function by regulating the ratio of ω‐3 to ω‐6 FAs in quinoa within the context of a balanced diet.

**TABLE 1:** Main FAs content of quinoa at different germination times.

FAs	Content (mg/100 g)				
	0 h	24 h	36 h	48 h	72 h
Oleic acid	2755.1 ± 62.0	2043.9 ± 88.1	1331.9 ± 19.2	1046.1 ± 34.8	545.1 ± 13.4
Linoleic acid	413.3 ± 13.2	330.7 ± 11.6	210.9 ± 1.2	182.9 ± 4.7	128.3 ± 3.5
Linolenic acid	32.2 ± 1.5	31.6 ± 1.6	18.5 ± 2.5	15.6 ± 0.6	12.9 ± 1.0
Palmitic acid	254.4 ± 21.8	188.1 ± 8.3	122.7 ± 1.0	108.9 ± 2.7	81.8 ± 1.3
Docosenoic acid	79.8 ± 3.3	79.3 ± 10.9	48.4 ± 6.9	38.7 ± 0.5	28.4 ± 0.3
Docosanoic acid	20.8 ± 2.0	19.6 ± 3.1	15.3 ± 1.3	13.2 ± 1.5	11.4 ± 1.8

### 3.2 Changes in Folate Content of Quinoa

**FIGURE 3:**
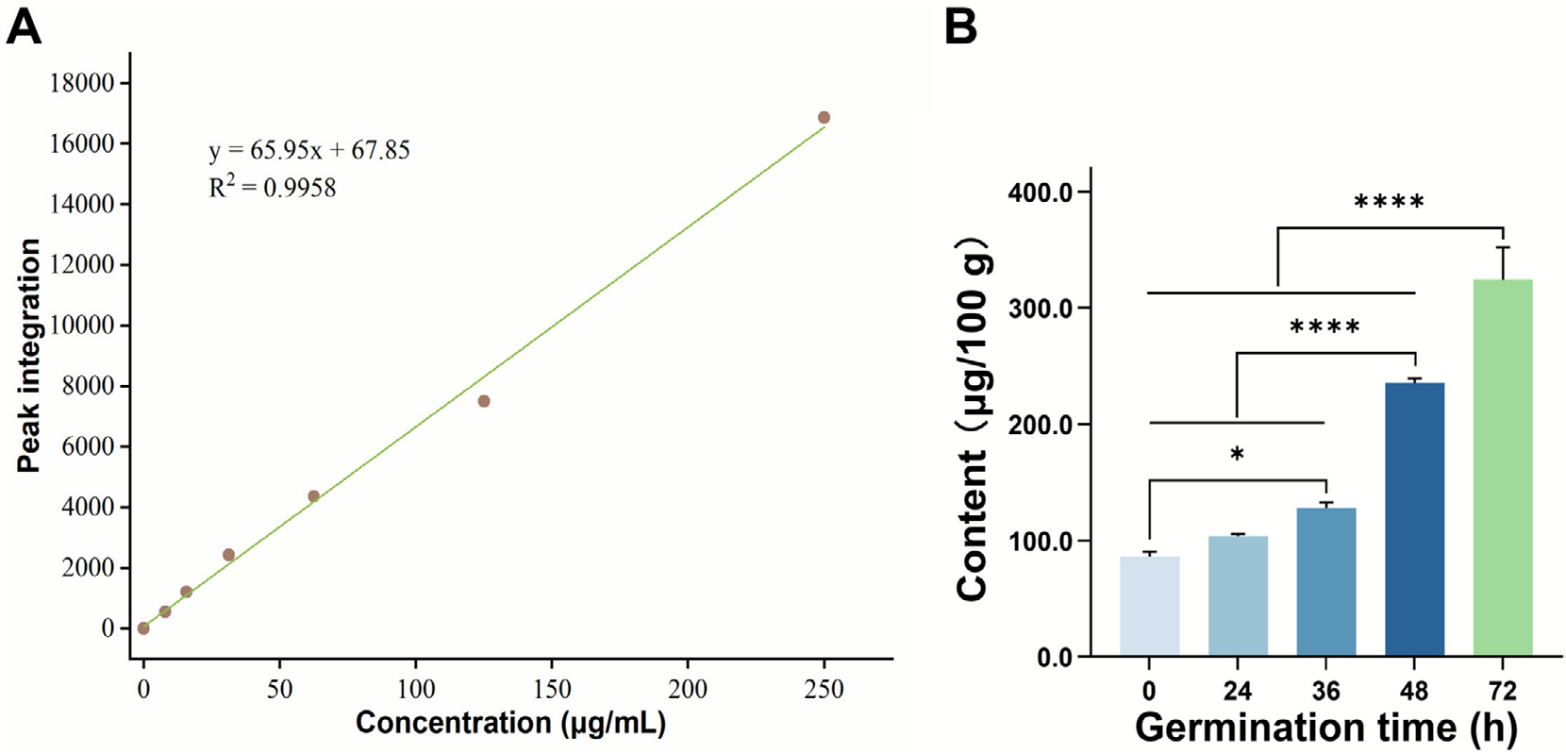
(A) Folate standard curve, and (B) changes in folate content at different germination times. (*p < 0.05, **p < 0.01, ***p < 0.001, ****p < 0.0001).

The standard curve of folate is shown in Figure 3A. The folate contents of germinated quinoa are shown in Figure 3B. When the germination time was 48 h, the folate content of the sample increased by 1.73 times, which was significantly different from that of ungerminated quinoa. At 72 h, the folate content was 349.63 μg/100 g sample, which was three times higher than that of ungerminated quinoa. The increase in folate content during germination is the comprehensive result of plant physiological activities, primarily involving the activation of biosynthesis and the transformation of metabolic pathways after seed dormancy is released. For example, the p‐ABA branch is crucial for the accumulation of folate, and the pterin branch is the key control point of the single‐carbon pool of the folate pathway (He et al. 2024). In addition, the use of red light radiation can enhance the accumulation of folate in wheat seedlings during germination (Xie et al. 2022). This method may also increase the folate content of sprouted quinoa.

For infants and young children, folate required for their early brain development is completely dependent on maternal supply. Sufficient folate can significantly reduce the risk of neural tube defects, such as spina bifida and anencephaly (Kakumoto et al. 2021). Comprehensive medical guidelines recommend that women without high‐risk factors should supplement 400–800 mg of folate daily, starting before pregnancy and continuing until the end of the first trimester (US Preventive Services Task Force et al. 2023). Based on the results of this study, pregnant women could meet their nutritional needs by consuming 200 g of sprouted quinoa products per day. However, considering the loss of folate in food during cooking, the actual amount needed may be higher. But this still means that germination can be an especially effective method for increasing the folate content of quinoa, making sprouted quinoa a promising option as a folate‐enhanced food to improve early brain health in babies. This strategy may be particularly important for economically underdeveloped areas and low‐income individuals, as it eliminates the need to purchase expensive folate supplements or folate‐rich foods, which are more expensive than quinoa.

### 3.3 Changes in Protein Content of Quinoa

**FIGURE 4:**
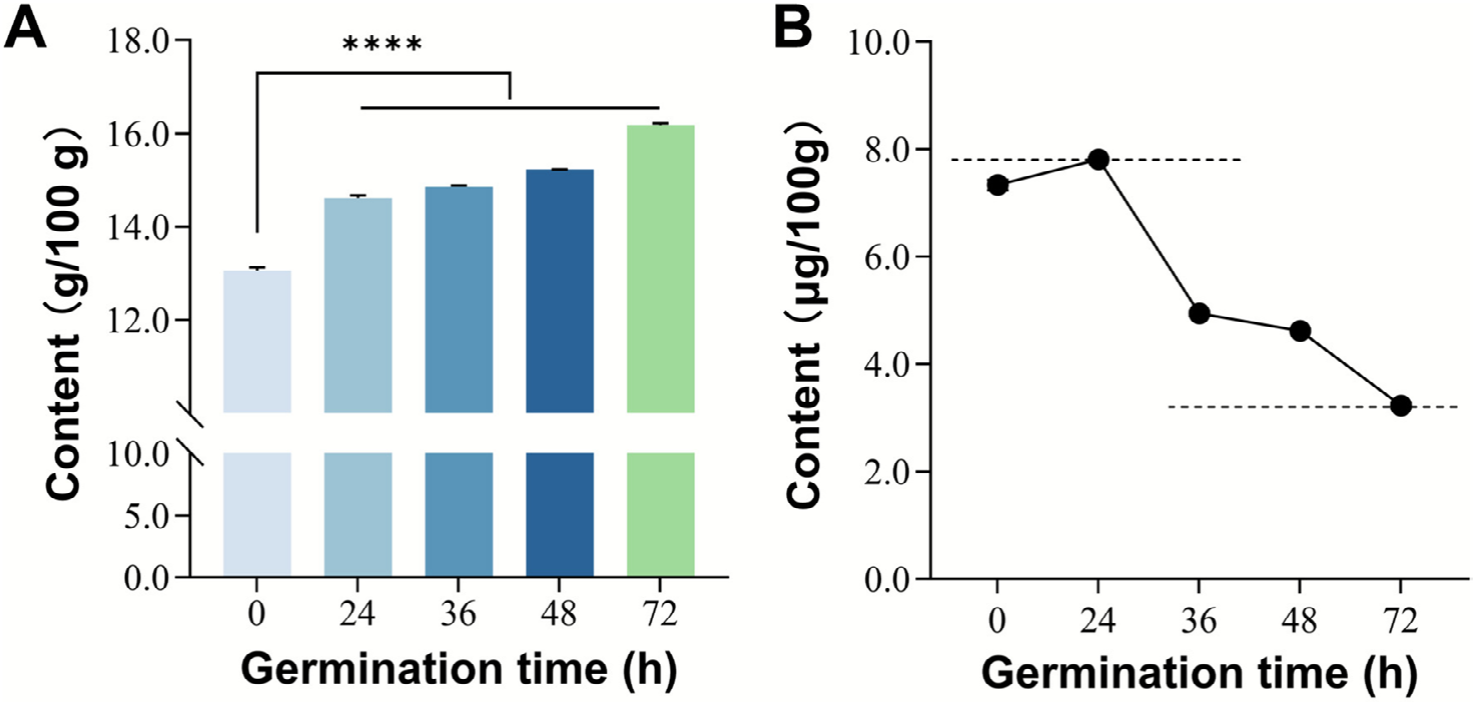
(A) Changes in crude protein content, and (B) Se content. (*p < 0.05, **p < 0.01, ***p < 0.001, ****p < 0.0001).

Although the Kjeldahl method can give the crude protein content, it cannot determine the specific amino acid content. It is still an efficient method for detecting protein content in food. As shown in Figure 4A, the protein content of germinated quinoa products at all stages was significantly higher compared to ungerminated quinoa. Especially, the crude protein content of each 100 g sample increased by 24.25% at 72 h. Firstly, this may be due to the synthesis of new functional proteins during the germination process. Research has demonstrated that to support the growth of radicles and plumules, seeds accelerate protein synthesis, with the rate of synthesis potentially surpassing that observed during the dormant period (Gumilevskaya et al. 2003). The newly synthesized proteins include amylase, dehydrogenase, and amino acid permease. In addition, the enzymatic conversion of storage proteins may also contribute to an increase in protein content. For example, cysteine proteases and aspartic proteases are activated after germination, leading to the hydrolysis of insoluble storage proteins, such as alcohol‐soluble proteins, into soluble peptides and free amino acids (Ghosh and Pal 2012). But based on the complex biochemical process involved in germination, the increase in small molecule proteins increases the protein absorbability, thereby improving the utilization rate of protein by the human body. Therefore, germination may be a potential method for enhancing the absorption and utilization of quinoa protein.

### 3.4 Changes in Mineral Content of Quinoa

Quinoa is abundant in minerals, such as Fe, Mg, K, and P. To facilitate analysis and discussion, we selected the top 10 elements, as shown in Table 2. Overall, most elements showed a trend of initially increasing and then decreasing throughout the germination process, with the germination time of 48 h being identified as the key peak period. Specifically, except for S and Cu, the contents of the remaining eight elements reached their maximum values 48 h after germination. The S content continued to increase until 72 h, rising from 137.04 to 194.55 mg/100 g, while the Cu content decreased slightly after peaking at 36 h. These changes are mainly due to the activation of the enzyme system in the quinoa during the germination process (Lintschinger et al. 1997). Phytase releases the originally bound mineral elements by decomposing the anti‐nutritional factor phytic acid, thereby improving the bioavailability of minerals (Badau et al. 2005; Maldonado‐Alvarado et al. 2023). At the same time, enhanced metabolic activity in the early stage of germination also promotes the mobilization and accumulation of minerals. The decrease in the content of various elements after 48 h may be related to the consumption of minerals during seedling growth, such as cell division and energy metabolism, or to resource reallocation. In particular, the significant increase in Ca and Fe highlights their key roles in cell wall formation and redox reactions (Huang et al. 2021). In addition, the continued accumulation of S may be attributed to the increased synthesis of sulfur‐containing amino acids in the late germination period (Mondal et al. 2022). Overall, germination significantly optimizes the mineral composition of quinoa, especially at 48 h, when the nutritional fortification effect is at its peak. This provides a scientific basis for improving its nutritional value as a functional food.

**TABLE 2:** Main mineral content of quinoa at different germination times.

Minerals	Content (mg/100 g)				
	0 h	24 h	36 h	48 h	72 h
K	554.04 ± 19.31	556.12 ± 12.78	579.11 ± 26.10	719.09 ± 17.38	637.01 ± 10.03
P	492.22 ± 7.22	533.97 ± 11.17	542.88 ± 8.01	595.09 ± 19.05	580.96 ± 14.24
Mg	216.23 ± 10.77	229.11 ± 3.14	238.09 ± 5.03	260.87 ± 18.80	252.00 ± 5.56
S	137.04 ± 3.19	169.02 ± 4.08	172.42 ± 11.01	183.65 ± 10.00	194.55 ± 8.30
Na	133.67 ± 6.07	112.04 ± 7.11	129.29 ± 4.07	184.01 ± 7.92	150.22 ± 3.01
Ca	41.72 ± 0.99	63.44 ± 1.07	65.61 ± 3.21	171.33 ± 24.35	104.05 ± 9.82
Fe	6.14 ± 0.20	6.20 ± 0.32	6.64 ± 0.21	10.8 ± 0.21	7.24 ± 0.42
Zn	2.82 ± 0.01	2.87 ± 0.03	2.99 ± 0.11	3.02 ± 0.09	2.79 ± 0.02
Mn	2.29 ± 0.02	1.98 ± 0.01	2.05 ± 0.01	2.84 ± 0.03	2.22 ± 0.03
Cu	0.63 ± 0.02	0.65 ± 0.03	0.67 ± 0.01	0.71 ± 0.01	0.65 ± 0.02

It is worth noting that the content and bioavailability of Fe, Cu, and Zn in sprouted quinoa significantly increase, effectively enhancing its role in promoting early brain development. During 48 h germination period, the Fe content reached a peak of 10.8 mg/100 g, an increase of 76% from the initial stage. The Zn content increased to 3.02 mg/100 g, representing a 7% increase. Meanwhile, the Cu content rose to 0.71 mg/100 g in 36 h, marking a 13% increase. Those three trace elements are crucial for the early brain health and development of infants and young children. Fe, as a key factor in oxygen transport and myelin synthesis, supports cognitive development; Zn ensures synaptic plasticity and hippocampal function; and Cu drives dopamine metabolism and antioxidant defense (Cheatham 2019; Morton et al. 2022). In addition, phytase decomposes phytic acid to release more easily absorbed free trace elements (Brinch‐Pedersen et al. 2007). Consequently, germination can enhance the release of trace minerals in quinoa and boost the levels of beneficial functional ingredients that support neural development.

As we all know, Se is a core component of a variety of Seleno‐based proteins, which are essential for the antioxidant defense system of the developing brain (Schweizer and Fabiano 2022). They can effectively remove free radicals, reduce oxidative stress damage, and protect the integrity of nerve cell membranes and DNA, thereby maintaining the stability and function of neuronal structures (Schweizer and Fabiano 2022). However, as shown in Figure 4B, the Se content decreased by 57% with germination time, dropping from 7.38 μg/100 g at 0 h to 3.2 μg/100 g at 72 h. This may be due to the loss of water solubility and metabolic transformation of Se during water immersion. Nevertheless, the effects of Fe, Zn, and Cu on early brain development far exceed those of Se, especially given the high incidence of Fe deficiency anemia in infants and the sensitivity of neurodevelopment (Hosseini et al. 2024). Therefore, the synergistic peaks of the three trace elements of Fe, Zn, and Cu in quinoa products with a germination time of 48 h can maximize the nutritional benefits, significantly improving quinoa's value in protecting brain health.

## 4 Discussion

This study shows that controlled germination can transform quinoa into a powerful functional food that benefits early brain development. After 24 h of germination, quinoa has just begun to germinate, resulting in minimal loss of FAs and a more balanced ratio of ω‐6 to ω‐3 FAs. As the germination time reaches 48 h, the fat content, which serves as the main energy source, drops rapidly. However, under the action of enzymes, the folate content of germinated quinoa increased by 1.73 times. In addition, the levels of Fe, Zn, and Cu, three important trace elements for early brain health of infants, reached a peak. Furthermore, the content of crude protein also increases significantly. Therefore, we believe that quinoa products with a germination time of 48 h are the most nutritious options for supporting early brain health.

Our innovation is the first comprehensive analysis of quinoa's nutritional profile over time, demonstrating that sprouting, as a biotechnology process, can effectively enhance quinoa's neuroprotective potential. This approach could help tackle the global challenge of malnutrition by providing a cost‐effective whole‐food alternative to synthetic supplements, particularly in prenatal and infant nutrition, where folate deficiency and anemia are prevalent and require scalable solutions. Although there are some limitations, such as unvalidated in vitro neuronal cell viability levels and in vivo bioavailability, future studies should use animal models to measure nutrient absorption and neurocognitive outcomes, optimize sprouting protocols, and conduct comparative analyses with different quinoa varieties. Nevertheless, this work uniquely connects food science to early brain health by establishing chrononutrition as a sustainable strategy. This study demonstrates that quinoa with a favorable FA ratio, high folate levels, and trace minerals meeting the recommended daily intake can be produced through simple germination, without the need for complex methods like genetic modification. This study positions sprouted quinoa as an innovative, cost‐effective approach to protect early brain health in infants, address neurodevelopmental disorders, and support the Sustainable Development Goals of global health equity.

## 5 Conclusion

Quinoa has great potential as a versatile food in the food industry, but its potential link to early brain health has not been studied. This study employed GC–MS and HPLC‐UV methods to comprehensively analyze the core nutrients of quinoa during a short germination time. This study showed that 48 h sprouting can significantly regulate the key bioactive components of quinoa, thereby maximizing its neurodevelopmental nutritional properties. Sprouting increased folate content by 1.73 times and protein content by 17.10%. Important trace elements, including Fe, Zn, and Cu increased by 75.9%, 6.4%, and 13.6%, respectively. This study confirms that germination is an effective method for regulating the nutritional levels of quinoa. Furthermore, the unique nutritional composition of germinated quinoa is crucial for early brain health. This study proposes a method to transform quinoa into a special food for protecting brain health without genetic modification or fortification. This work offers a scalable strategy to combat neurocognitive disorders while providing a food‐based solution for low‐income groups.

## Author Contributions

**Boyuan Li:** investigation, data curation, writing – original draft, visualization. **Ting Wang:** writing – review and editing, visualization. **Muhui Wang:** data curation, visualization. **Yueying Zhang:** software, visualization. **Pak‐Ho Wong:** data curation, visualization. **Yi Wang:** conceptualization, validation, resources, supervision, project administration, writing – review and editing, writing – original draft, funding acquisition, visualization.

## Funding

This work was supported by Shenzhen Virtual University Park (2021Szvup133).

## Conflicts of Interest

The authors declare no conflicts of interest.

## Data Availability

The data that support the findings of this study are available from the corresponding author upon reasonable request.
